# A Few Observations and Experiences in Porcelain Work

**Published:** 1906-12

**Authors:** M. D. Huff

**Affiliations:** Atlanta, Ga.


### A FEW OBSERVATIONS AND EXPERIENCES IN PORCELAIN WORK.

By Dr. M. D. Huff, Atlanta, Ga.

Georgia State Dental Society, 1906.

The object of this short paper is not to teach this intelligent body porcelain work, but it is written in the hope that some thought may be suggested and be brought out in the discussion that may follow.

And, too, the special cases are reported with the idea that perhaps some one here may have a case of his own at home that might be made easy in a similar manner.

First. We will take as granted that we have an approximal cavity prepared, and wish to make a matrix. The matrix to be used should be platinum, because it can be used at least one-third as thick as gold, and yet be more rigid and thus resist distortion in removing from cavity or in fusing of porcelain. This is true whether low or high fusing body is used.

Now, place platinum 1-1000 in position, bend down surplus over tooth labially and lingually and pull taut over this a strip of tightly woven tape and with a burnisher, the matrix is quickly adapted. In labial cavities, cotton or punk and an automatic mallet is used to advantage by using orange wood as a plugger, or pack in gum camphor, if you please.

As to the Selection of Color.—It is rarely the case that we have any one color that will match the natural tooth. I have often used R. S. & T. in one inlay with very good results by baking one color darker, and then adding the others. Had one case where it was a little too light, so I set inlay with Ames brown cement and got a perfect match. All of us have had trouble with color changing when set.

The plan I adopted is, to set in oxide of zinc, mixed in water first to test color effect. An idea secured in a journal.

In baking, I always time the furnace. One can soon become expert at that, and time according to heat of furnace. It is much better than looking into the furnace, even with glasses.

Preparation for Setting.—It was suggested to me some time ago to grind the surface of an inlay to make the cement stick. I tried it in *four* labial cavities and incisors a couple of months ago or so, and *four* of them have been reset already. Now, I go back to the old rule and etch or undercut every one. Only etching small fillings.

As to Setting.—I usually slightly undercut cavity and use either Ames or Harvard inlay cement.

One of the first cases I ever attempted in porcelain, was that of a young lady about eighteen years of age, who unfortunately had what we know as Hutchinson's teeth. We undertook to give a proper contour to incisal edges of her teeth by grinding o a portion of cutting edge and then cutting a groove and form-in matrix, then drilling two holes for reception of short staple of twenty gauge Iridio platinum wire. Matrix was removed in each, an impression of modeling compound a model of equal parts of plaster and fine silex made.

Case 2.—Mr. C, aged fifty, had chemical erosion of upper centrals and laterals. The extent of central cavities was such that each one embraced a portion of labial mesial and distal surfaces.

The bottom of the cavity is about half way through the tooth, the nerve having receded from age and irritation. The incisal border of cavities come within two or three lines of cutting edge of teeth. By filling partially with cement and simply cutting away sufficient dentine to give proper cavity margin, very good results were obtained.

Case 3.—Mr. E presented himself, having a large amalgam filling in the labial surface of the root of an upper left central. The cavity extended from the cervical portion of the tooth up to two-thirds of the length of the root, the gum having receded that far. In other words, the inlay was the restoration of the anterior two-thirds of the root. Inlay was made in usual way, but required five bakes with the additional precaution of reburnishing after each bake and necessitating the breaking of the inlay twice on account of shrinkage. But finally the fit was good and results pleasing. This is a case you would have to see to appreciate.

Inasmuch as the gum was irritated, spongy, it has now assumed normal color and proportions, to say nothing of the æsthetic effect.

Case 4.—Miss W split off almost half buccal portion of root of first bicuspid upper. A staple of nineteen gauge Iridio platinum wire was made and a disc of thirty-two gauge platinum used. A porcelain crown constructed, the disc was burnished so as to form matrix and the last portion of the root restored with high fusing body.

The gum is normal, results satisfactory.

Discussion.

Dr. W. H. Weaver:—My experience I know has been limited, and I was one of the first dentists in the state to try it, and I think the methods used by Dr. Huff are correct. We read in quite a number of journals that it was best to use it in layers, as he suggested, to get the correct color. I have not been able to do this. I have not been able to get the color of one layer to show through another with S. S. White's porcelain, and so I have used Brewster's porcelain, and have in that way been able to procure the shades desired.

Another point in the paper I like is that of testing for color by investing in wet oxide of zinc before the piece is finished and placed in the mouth. I think that is a capital idea. Another valuable point which is not referred to in the paper, but which I have found by experience, is not to undertake to bake porcelain until it has been subjected to pressure. He speaks of the use of platinum instead of gold for the matrix, which I think correct; but I have never been able to get adaptation by the use of the wood points. In the final burnishing of the matrix in the cavity there is a possibility of changing its form.

Dr. H. H. Johnson:—I consider the advent of porcelain one of the greatest additions to our methods of practice. It is great for these reasons. Some of us heard what Dr. Weaver alluded to concerning the irritation at the points about the gum margins of teeth. We must come to that. The evil effects of these conditions are so apparent that we must make efforts in practice whereby this can be avoided. And then again, porcelain being a perfect non-conductor it will be less liable to irritate dentine or kill nerves than any metallic filling we have. Therefore the tooth takes kindly to it; and then the esthetic features must not be lost sight of, building up shell walls and broken down teeth with beautiful white porcelain that blends in color to the original tooth, making a beautiful esthetic effect.

Dr. H. W. Walker:—I wish to endorse Jenkins' low fusing body. I used White's high fusing body for four years experimenting, and I now have some large contour fillings with high fusing body that have been in there about three years. I saw one last week, and it is apparently as good today as it was then. I used Ames' cement, and I have had the same experience with high fusing body that Dr. Johnson speaks of. I had to run it up to the fifth button on Hammond's furnace, and let it stay one and three-quarters to two minutes in the furnace. I bought some of Jenkins' low fusing body and was timid about using it for fear it would not stand. I put in some about six months ago, which I have seen recently, and they are doing well. It will fuse at lower temperature. I use gum camphor in burnishing my matrix, and then burned it out and do not invest it at all.

Dr. DeLos Hill:—I am very glad Dr. Walker has brought out the thought of using gum camphor in matrices. I had been experimenting with porcelain three or four years before I ever put a filling in the mouth and that is the method of Dr. F. C. Houseworthy, of Philadelphia, who has been working with porcelain for the past fourteen years now. He was demonstrating last year for S. S. White Dental Co. all over the county and put porcelain fillings in the mouth with the patient remaining in the chair just as you would with gold fillings; on the last burnish, as you might call it, packing gum camphor in the matrix, removing the matrix and burning it out, he claiming that the gum camphor holds the matrix in perfect condition, and that has been my experience. First he absorbs all of the moisture with blotting paper if you please, and afterwards by tapping and with his burnisher. It is very obvious that he gets a great deal more dense filling by the pressure on that porcelain than you would if you simply let it go to place by the tapping, as I presume most of you do. He finishes that filling and sets it by at one side.

On the subject of bodies, most of us have had it instilled into us that high fusing porcelain is the only porcelain. I beg to say that I think the middle fusing, S. S. White porcelain, is the best, replacing other high fusing porcelain. You can get high fusing, low fusing or medium fusing porcelain. Most people want high fusing. To those using high fusing porcelain, I would suggest you try this medium fusing, which I am using almost entirely now. I think you will get better results than with the high fusing.

Dr. H. H. Walker:—Of course the current may vary at different places, but with a little practice and by making a few trial bakings of the body, one can soon bake the inlays by timing and get perfect results. I have had experience with only two of the bodies, viz: White's high fusing and Jenkins' low fusing bodies. The latter I much prefer; the high fusing burns out the muffle, consumes much time besides the intense heat being very disagreeable to the eyes. To fuse the White's high fusing body on my Hammond furnace, it requires one minute to bake each, the first, second, third and fourth buttons, and two minutes on the fifth button. Whereas, with the Jenkins' low fusing body, it only requires one minute on the first button.

While speaking of porcelain, I wish to mention a way of perfectly adapting a Logan crown by the use of porcelain; especially is this method of great benefit when we cannot get a Logan large enough around the neck to cover the end of the root. After the root has been cut down to the gum margin and prepared in the usual manner, cut a round piece of 24-k gold 36 gauge just the size of the root abutment and place this against the root and swage it to position by tapping gently a soft pine stick against it, but use a small quantity of wet cotton over the gold between the end of stick. This gives a perfect adaptation of the gold disk; now take the Logan crown, which has already been ground approximately to fit, and mix a small quantity of the body, color to match the crown and place on the body of crown right into position; then withdraw and the gold will come away with it. Now we have the porcelain body occupying the space between the imperfectly fitting crown and the perfectly fitting gold disk, and after fusing this we have a perfect adaptation, and also build out the crown to desired size at the point of contract.

Dr. Jackson:—I have had very little experience with porcelain, but that has been altogether with Jenkins' body, and I

want to say to those who would like to take up the use of porcelain, and who have not the electric current, whether you have or have not, and do not want to go to the expense of investing in an electric furnace that you can fuse Jenkins' body all right with the ordinary gas or gasolene blow pipe if you please and if you want to experiment with it in that way, all you want is Jenkins' cup of investment material, and a few bottles of porcelain body.

